# Overall survival according to timing of immune checkpoint inhibitors administration in patients with advanced cancer: results from a large single-centre cohort analysis

**DOI:** 10.1038/s41416-026-03494-y

**Published:** 2026-07-18

**Authors:** Tommaso Bosetti, Oliver John Kennedy, Raffaele Califano, Tom Waddell, Rob Metcalf, Sophia Kreft, Rebecca Lee, Paul Lorigan

**Affiliations:** 1 The Christie NHS Foundation Trust, Manchester, UK; 2 University of Pavia, Pavia, Italy; 3 University of Manchester, Manchester, UK

**Keywords:** Cancer immunotherapy, Immunotherapy, Lung cancer, Melanoma, Renal cancer

## Abstract

**Background:**

Immune checkpoint inhibitors (ICIs) have revolutionised cancer treatment. Previous studies suggested that early time-of-day administration is associated with better outcomes. We performed a retrospective study in patients treated at a large tertiary cancer centre.

**Methods:**

Patients receiving ICIs between January 2018 and December 2023 were grouped according to whether they had ≥50% (“late group”) or <50% (“early group”) of cycles after the median time of all treatments. The endpoint was overall survival (OS). Hazard ratios (HRs) were estimated using Cox models with and without time-dependent variables.

**Results:**

2631 patients with advanced/metastatic lung cancer (45%), melanoma (23%), renal carcinoma (18%), head and neck (9%), and urothelial cancer (5%) were included. Median age was 68.2 years and median infusion time was 12:49 h. At a median follow-up of 32 months, median OS was 13.1 (95% CI 11.8–14.4) vs 21.4 (19.8–24.5) months for late and early group. Late group was associated with shorter OS on both the standard analysis (HR 1.48, 1.33–1.63) and the time-dependent model (HR 1.30, 1.16–1.44).

**Conclusions:**

This study provides further evidence in favour of early ICIs administration with a large sample size and a time-dependent model, which reduces the risk of immortal time bias. Data from randomised trials are required to confirm these findings.

## Background

Immune checkpoint inhibitors (ICIs) have revolutionised outcomes for many patients across a wide range of tumours [1]. These drugs work by enhancing the immune system’s ability to recognise and eliminate cancer cells. However, there is significant variability in efficacy between different tumour types and individuals with the same tumour [2]. Factors such as tumour burden and microenvironment, as well as patient characteristics, can influence the response to treatment [3, 4].

Circadian rhythms modulate the activity of the immune system throughout the day [5]. This includes differences in immune cells proliferation, leukocyte migration, antigen recognition, and cytokine circulation [6]. There is evidence, including randomised trials, that vaccinations have worse outcomes when given in the afternoon [7–9]. Chronotherapy (aligning time of treatment with circadian rhythms) has been shown to reduce chemotherapy and radiotherapy toxicity [10, 11].

More recently, an increasing number of retrospective studies suggested that time of day of immunotherapy administrations may play a role in treatment efficacy [12–23]. Overall, these suggest a consistent improvement in overall survival (OS), progression-free survival (PFS), and response rate when most ICIs infusions are given early during the day in melanoma, non-small cell lung cancer (NSCLC), renal cell carcinoma (RCC), urothelial, oesophageal, gastric, and biliary tract cancer. However, in most of these studies “early” versus “late” treatment groups were defined retrospectively, allowing future treatment exposure to influence group assignment and potentially introducing immortal time bias. A recent small phase III trial in 210 patients with advanced NSCLC reported that administration of the first four cycles of immunochemotherapy before 15:00 h was associated with improved PFS and OS [24].

We report the impact of time of treatment with ICIs in a large patient cohort across a number of tumour types, using more robust methods to minimize confounding factors.

## Methods

We performed a single-centre retrospective cohort study at The Christie NHS Foundation Trust, Manchester, UK, a large tertiary cancer centre treating over 60,000 patients a year. We included all ICI-naïve adult patients (≥18 years) who received standard-of-care ICIs (alone or in combination with chemotherapy or tyrosine kinase inhibitors [TKIs]) as first or subsequent-line treatment for unresectable or metastatic NSCLC, small cell lung cancer (SCLC), melanoma, RCC, urothelial, and head and neck (H&N) cancer between January 2018 and December 2023.

Patient, treatment, and disease characteristics were obtained from electronic medical records. The start time of each treatment administration was extracted from treatment prescription software. Patients were grouped by whether they received ≥50% (“late group”) or <50% (“early group”) of cycles after the median time of all patients’ treatments. The primary outcome was OS (median and one-year), defined as time to death from any cause from first ICI administration.

Median (interquartile range [IQR]) values were calculated for continuous variables, and proportions (percentages) for categorical variables. OS curves for early and late groups were generated using the Kaplan-Meier method and compared with a log-rank test. HRs were estimated using Cox proportional hazards regression, adjusting for age, sex, Eastern Cooperative Oncology Group performance status (ECOG PS), cancer type, and treatment. Analyses were conducted using both a standard Cox proportional hazards model and a Cox model with a time-dependent covariate for the treatment group. The proportional hazards assumption was verified using scaled Schoenfeld residuals. In the standard Cox model, patients were assigned at baseline to the early or late group based on whether at least 50% of their total infusions occurred before or after the predefined cut-off time. In the time-dependent Cox model, assignment to the early or late group was updated dynamically at each cycle—i.e., patients were classified as late if ≥50% of their treatments up to that point had occurred after the cut-off. This allowed for reassignment as treatment progressed and ensured that group assignment was based only on past treatment cycles, mitigating immortal time bias. As the first cycles are likely to represent the most important, we also conducted a sensitivity analysis where patients were allocated based on exposure at three months from the first administration. To assess the robustness of the findings to the selected time-of-day cut-off, sensitivity analyses were performed using alternative thresholds ranging from 10:00 h to 17:00 h. All analyses were performed using R Software (v4.4.0). All *p* values reported were two-sided, and a level of 0.05 was considered statistically significant.

## Results

### Patient characteristics

**Fig. 1:**
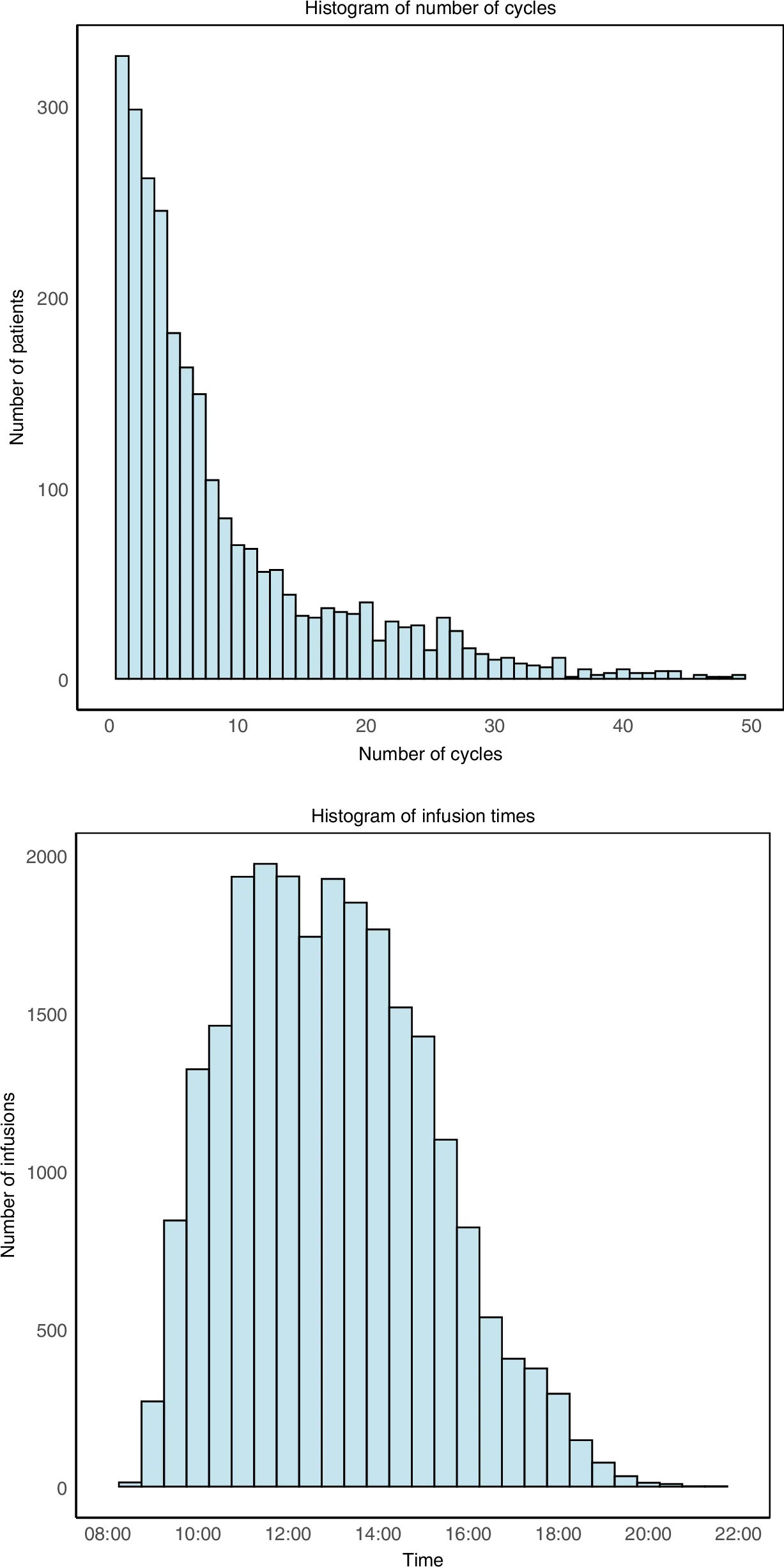
Histograms showing distribution of number of cycles and treatment times in the entire cohort.

A total of 2631 patients were included. Most patients were male (61%) and most common ECOG PS was 0 (56%). Median age at ICIs initiation was 68.2 years (IQR 15.2). Cancer types were NSCLC (39%), SCLC (6%), melanoma (23%), RCC (18%), H&N (9%), and urothelial (5%). Patients received ICIs alone (72%), ICIs plus chemotherapy (22%), and ICIs plus TKIs (6%). There were 1327 (50%) patients in the early group and 1304 (50%) in the late group. Baseline patient and treatment characteristics are shown in Table 1. Median follow-up was 32 months (95% CI 30–34 months). Median number of infusions per patient was six (range 1–139, IQR 10), and median infusion time was 12:49 h (Fig. 1). The proportion of treatments after the median for early and late groups is shown in Supplementary Fig. S1.

**Table 1:** Patient and treatment characteristics.

	All patients *N* = 2631 (%)	Early group *N* = 1327 (%)	Late group *N* = 1304 (%)
Sex			
Female	1029 (39)	497 (37)	532 (41)
Male	1602 (61)	830 (63)	772 (59)
Age			
<50	196 (7)	85 (6)	111 (9)
50-64	867 (33)	413 (31)	454 (35)
>65	1568 (60)	829 (62)	739 (57)
Median age – years (IQR)	68.2 (15.2)	68.9 (15.0)	67.7 (15.6)
ECOG Performance Status			
0	1470 (56)	748 (56)	722 (55)
1	1006 (38)	506 (38)	500 (38)
2+	118 (4)	57 (4)	61 (5)
Unknown	37 (1)	16 (1)	21 (2)
Median number of cycles (IQR, range)	6 (10, 1–139)	8 (13, 1–139)	5 (7, 1–72)
Disease and treatment			
NSCLC			
First line atezolizumab or pembrolizumab	384 (15)	221 (17)	163 (13)
Second line atezolizumab or pembrolizumab	218 (8)	119 (9)	99 (8)
Pembrolizumab or atezolizumab plus chemotherapy	424 (16)	206 (16)	218 (17)
SCLC			
Atezolizumab plus chemotherapy	158 (6)	66 (5)	92 (7)
Melanoma			
Ipilimumab plus nivolumab	325 (12)	157 (12)	168 (13)
Nivolumab or pembrolizumab	271 (10)	155 (12)	116 (9)
RCC			
Avelumab or pembrolizumab plus TKI	163 (6)	88 (7)	75 (6)
Nivolumab	155 (6)	68 (5)	87 (7)
Ipilimumab plus nivolumab	145 (6)	46 (3)	99 (8)
H&N			
Nivolumab or pembrolizumab	249 (9)	134 (10)	115 (9)
Urothelial			
Atezolizumab or pembrolizumab	139 (5)	67 (5)	72 (6)

Early group ≤ 50% administrations after the median time. Late group ≥/= 50% administrations after the median time.

*IQR* Interquartile Range, *ECOG* Eastern Cooperative Oncology Group, *NSCLC* non-small-cell lung cancer, *SCLC* small-cell lung cancer.

### Overall survival according to time of day of treatment

**Fig. 2:**
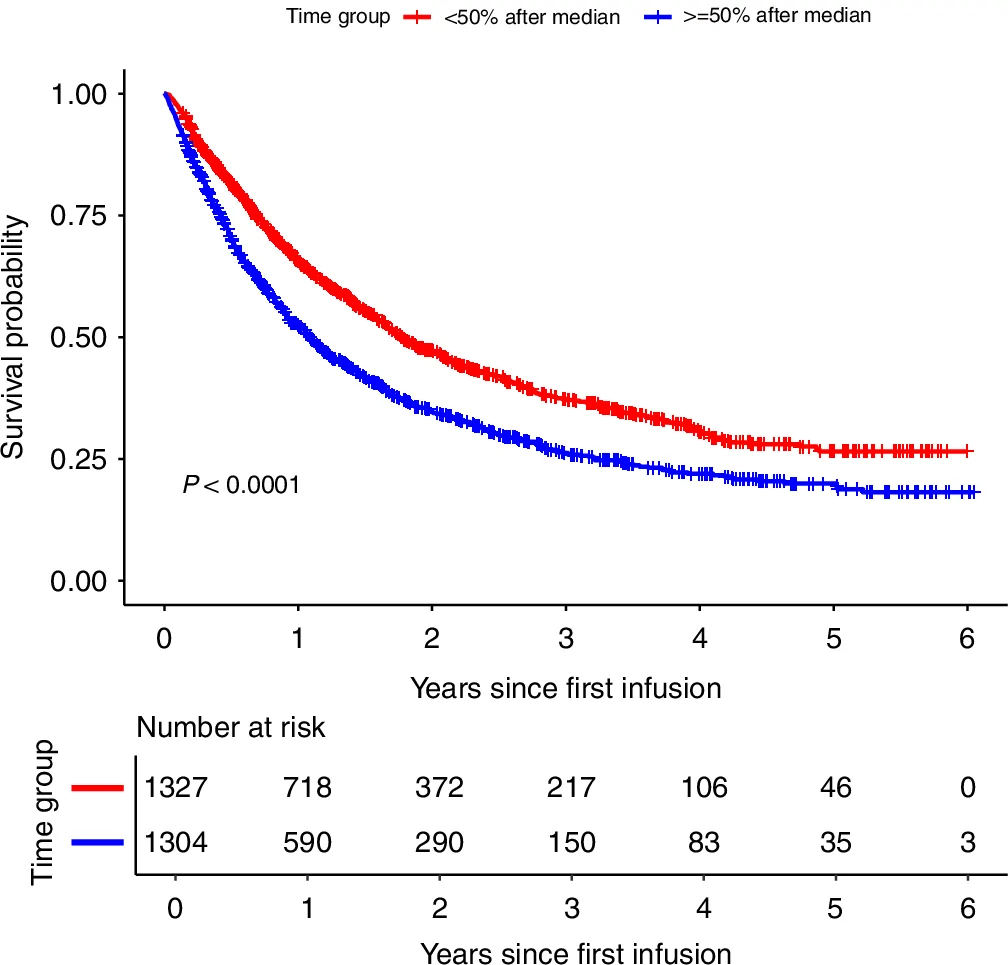
Kaplan-Meier curves for patients who received <50% vs. ≥50% infusions after the median time of infusion (entire cohort).

In the entire cohort, patients in the early group had a statistically significant longer OS compared to those in the late group (median OS 21.4 [95% confidence interval (CI) 19.8–24.5] vs 13.1 [95% CI 11.8–14.4] months; one-year OS 65.7% [95% CI 63.0–68.4] vs 52.5% [95% CI 49.7–55.4]) (Table 2). Kaplan-Meier curves are shown in Fig. 2. On multivariate analysis with the standard Cox model, following adjustment for age, sex, ECOG PS, and treatment, patients in late group had a higher risk of death compared to those in early group (hazard ratio [HR] 1.48, 95% CI 1.33–1.63). The association with OS remained significant when the time-dependent Cox model was applied (HR 1.30, 95% CI 1.16–1.44) (Table 3). The sensitivity analysis based on exposure at three months showed no difference between groups in the entire cohort with the standard model (HR 1.09, 95% CI 0.98–1.20, *p* = 0.109) and a higher risk of death for the late group with the time-dependent model (HR 1.14, 95% CI 1.02–1.26, *p* = 0.016) (Supplementary Tables S1–S2). The results of the sensitivity analysis using alternative time-of-day thresholds are shown in Supplementary Table S3.

**Table 2:** Overall survival according to timing of administration for the whole duration of treatment.

	Early group		Late group		
	mOS (95% CI) – months	1-year OS (95% CI) – %	mOS (95% CI) – months	1-year OS (95% CI) – %	*p* value
All patients	21.4 (19.8–24.5)	65.7 (63.0–68.4)	13.1 (11.8–14.4)	52.5 (49.7–55.4)	<0.001
NSCLC					
First line atezolizumab or pembrolizumab	24.4 (18.8–33.1)	65.7 (59.3–72.8)	11.8 (8.6–17.5)	49.4 (42.3–57.7)	0.001
Second line atezolizumab or pembrolizumab	16.6 (10.7–20.6)	54.1 (45.3–64.7)	8.4 (6.9–10.1)	37.8 (29.8–48.1)	0.002
Pembrolizumab or atezolizumab plus chemotherapy	17.2 (14.6–19.9)	65.5 (59.1–72.5)	16.3 (12.7–20.1)	59.0 (52.4–66.5)	0.177
SCLC					
Atezolizumab plus chemotherapy	11.6 (9.6–14.3)	48.5 (37.7–62.3)	9.8 (8.4–13.5)	41.8 (31.7–55.1)	0.363
Melanoma					
Ipilimumab plus nivolumab	NR (NR–NR)	78.5 (72.1–85.6)	22.4 (14.0–NR)	60.5 (53.3–68.6)	<0.001
Nivolumab or pembrolizumab	43.1 (23.4–52.6)	76.8 (69.6–84.8)	21.1 (14.9–33.3)	62.4 (54.8–71)	0.013
RCC					
Avelumab or pembrolizumab plus TKI	NR (NR–NR)	89.5 (83.3–96.3)	35.2 (23.9–NR)	70.6 (60.0–83.2)	0.064
Nivolumab	17.0 (12.3–26.5)	61.0 (50.6–73.4)	15.0 (10.8–22.1)	55.2 (45.2–67.5)	0.289
Ipilimumab plus nivolumab	39.2 (34.0–NR)	96.0 (90.8–100)	26.1 (18.2–NR)	64.6 (55.4–75.3)	0.013
H&N					
Nivolumab or pembrolizumab	9.8 (8.3–12.5)	40.9 (32.8–50.9)	8.0 (5.8–12.1)	40.4 (32.1–50.7)	0.24
Urothelial					
Atezolizumab or pembrolizumab	8.0 (6.6–15.8)	42.5 (31.8–56.6)	7.2 (5.7–10.7)	30.5 (20.8–44.6)	0.288

Early group ≤ 50% administrations after the median time. Late group ≥/= 50% administrations after the median time.

*OS* overall survival, *NSCLC* non-small-cell lung cancer, *SCLC* small-cell lung cancer, *RCC* renal cell carcinoma, *H&N* head and neck cancer, *TKI* tyrosine kinase inhibitors.

**Table 3:** Multivariable Cox proportional hazard regression of overall survival according to timing of administration for the whole duration of treatment.

	Overall survival HR (95% CI) for late group vs. early group			
	Standard Cox model	*p* value	Time-dependent Cox model	*p* value
All patients	1.48 (1.33–1.63)	<0.001	1.30 (1.16–1.44)	<0.001
NSCLC				
First line atezolizumab or pembrolizumab	1.45 (1.12–1.88)	0.005	1.28 (0.97–1.69)	0.084
Second line atezolizumab or pembrolizumab	1.52 (1.13–2.05)	0.005	1.45 (1.06–1.97)	0.020
Pembrolizumab or atezolizumab plus chemotherapy	1.20 (0.93–1.54)	0.165	1.14 (0.88–1.48)	0.310
SCLC				
Atezolizumab plus chemotherapy	1.19 (0.81–1.74)	0.381	1.11 (0.75–1.67)	0.597
Melanoma				
Ipilimumab plus nivolumab	1.98 (1.37–2.87)	<0.001	1.24 (0.82–1.87)	0.310
Nivolumab or pembrolizumab	1.49 (1.06–2.09)	0.020	1.19 (0.83–1.71)	0.353
RCC				
Avelumab or pembrolizumab plus TKI	2.17 (1.19–3.98)	0.012	1.40 (0.73–2.68)	0.306
Nivolumab	1.21 (0.82–1.78)	0.348	1.35 (0.90–2.05)	0.150
Ipilimumab plus nivolumab	1.89 (1.10–3.24)	0.020	1.35 (0.73–2.51)	0.341
H&N				
Nivolumab or pembrolizumab	1.16 (0.86–1.56)	0.324	1.07 (0.78–1.46)	0.670
Urothelial				
Atezolizumab or pembrolizumab	1.29 (0.87–1.93)	0.209	1.30 (0.84–2.02)	0.237

Early group ≤ 50% administrations after the median time. Late group ≥/= 50% administrations after the median time.

*HR* hazard ratio, *NSCLC* non-small-cell lung cancer, *SCLC* small-cell lung cancer, *RCC* renal cell carcinoma, *H&N* head and neck cancer, *TKI* tyrosine kinase inhibitors.

The subgroup analysis showed that association with OS was not significant for all individual regimens. In the standard Cox model, this was significant for most ICI-alone regimens (*N* = 1886) and for the only TKI containing regimen (*N* = 163), whereas there was no association in any chemotherapy-containing regimens (*N* = 582). The highest benefit for early treatment was seen with combination immunotherapy (ipilimumab plus nivolumab for melanoma and RCC—HR 1.98 [95% CI 1.37–2.87] and 1.89 [95% CI 1.10–3.24], respectively) and with immunotherapy plus TKI for RCC (HR 2.17 [95% CI 1.19–3.98]). Even when not significant, with the standard Cox model the estimates for individual regimens were all suggestive of a worse outcome when most ICIs infusions were given after the median time. Kaplan-Meier curves for individual regimens are available in the Supplementary Figs. S2–S5.

## Discussion

To date, this is the largest study to analyse the time-of-day impact of ICIs on OS. Among 2631 patients with a range of advanced cancers treated at the same centre, receipt of most cycles later during the day was associated with worse OS (HR 1.48, 1.33–1.63). This was validated using a time-dependent Cox model, which allowed for dynamic group assignment when the proportion of early versus late treatments changed during follow-up.

Despite the benefit not reaching statistical significance for all regimens, the direction of the effect estimates was suggestive of worse outcomes with late administration, and the lack of significance may be explained by smaller sample sizes and possible imbalances in baseline patient characteristics. Interestingly, in the subgroup analysis with the standard Cox model, within the same tumour type the HRs were greatest with combination ICIs regimens (ipilimumab plus nivolumab for melanoma and RCC—HR 1.98 [1.37–2.87] and 1.89 [1.10–3.24], respectively), than with single-agent ICIs. Conversely, there was no association for chemotherapy-containing regimens. This suggests that the drug associated with the ICI backbone (chemotherapy, TKI, or another ICI) might play a role in enhancing or diminishing the effect of time of administration. Furthermore, although this was not addressed in our study, the treatment indication—adjuvant versus metastatic—may also be critically important. Two unplanned retrospective analyses of large, prospective randomised trials of adjuvant ICIs in resected high-risk melanoma showed no impact of time of treatment administration [25, 26].

Overall, our results align with growing retrospective evidence of a possible circadian impact on ICIs' efficacy. A meta-analysis of the first 13 studies by Landré and colleagues found that earlier administration of ICIs was associated with significantly improved OS and PFS in 1663 patients with several advanced cancers (HR 0.50 [95% CI 0.42–0.58, *p* < 0.00001] and 0.51 [95% CI 0.42–0.61, *p* < 0.00001], respectively) [27]. More recently, a review of 31 retrospective analyses (including the 13 previously discussed) in a wide range of cancer types and ICIs found that only one study showed longer PFS for patients treated “late”, whereas in the vast majority of studies the outcomes were improved in those treated earlier in the day [28]. In the first, small randomised phase III trial, Zhang and colleagues found that infusion of the first four cycles of chemoimmunotherapy before 15:00 h was associated with improved PFS and OS compared to infusion after 15:00 h in a cohort of 210 patients with advanced NSCLC [24]. However, post-publication concerns regarding inconsistencies between the trial registration, protocol, and reported findings warrant cautious interpretation pending further clarification.

There are several potential mechanistic explanations for these results overall. For instance, tumour infiltrating CD8 + T cells—the key components of anticancer immunity—show oscillations in quantity and quality across the day in mice and humans [29]. In the context of ICIs, in mouse models, treatment with anti-PD-L1 during the early active phase, when myeloid-derived suppressor cells and PD-L1 expression peak, stimulated immune infiltration and inhibited tumour growth [30].

Our study contributes to the current evidence with several methodological strengths. The sample size is the largest presented so far and patients with a variety of cancer types and treatments were included. We adjusted for major prognostic factors, such as age, sex, and ECOG PS. Analyses were conducted using both a standard Cox proportional hazards model and a Cox model with a time-dependent covariate, which minimizes the risk of immortal time bias. This can occur in retrospective studies when group assignment is based on future events (the timing of treatment cycles), making patients receiving fewer cycles (usually a marker of poor prognosis) more likely to be classified into “late” groups—either due to a single skewed late infusion when late-day cut-offs are used (Supplementary Table 3), or because early cycles are more likely to be scheduled later in the day for logistical reasons, whereas subsequent cycles are typically planned in advance and occur earlier. Some studies have addressed this using landmark analyses, where patients are grouped based on the proportion of early treatments at a fixed time (e.g. two months after their first treatment), with survival then compared from that point. However, this approach also has limitations, as it excludes patients who die before the cut-off.

However, this study also has a number of significant limitations. We did not have details on key tumour specific prognostic factors (e.g. stage, LDH, PD-L1 expression, BRAF status, IMDC score, subsequent treatment, etc). In addition, we were not able to control for many other potential biases. Symptomatic patients and those with lower income are more likely to require hospital transport and may, therefore, be less likely to be given an early morning appointment for treatment. Also, while most patients follow a nurse-led review protocol prior to their infusions, frail patients, those tolerating treatment less well or more symptomatic may have delays due to the need for medical review before treatment administration.

There is ongoing debate and uncertainty around the basis for any circadian effect on ICIs, given the long half-life and high receptor occupancy [31–34]. There is an argument that only the time of first administration would be important, yet responses evolve over time and with different patterns between individual patients. Further retrospective studies are unlikely to answer these outstanding questions. A further small, randomised phase II study is underway in which 99 metastatic melanoma patients will be randomised to ipilimumab plus nivolumab between either 8:00-11:00, 11:00-14:00, or 14:00-17:00 h, however this will not be practice changing [35]. A large randomised trial controlling for the major patient, disease and organisational confounding factors is needed to clarify whether chronotherapy influences ICI efficacy [36]. The magnitude of the benefit seen in the retrospective studies for what is largely a cost and toxicity neutral intervention justifies such a trial. Securing the support of patients will depend on effective communication of the ongoing equipoise around this question.

In conclusion, our findings suggest a significant survival benefit when ICIs are administered early in the day. However, a large prospective randomised trial is required to provide a definitive answer.

## Supplementary information

Supplementary material

## Data Availability

The datasets generated during and/or analysed during this study are not publicly available due to privacy restrictions.

## References

[1] Ribas A, Wolchok JD. Cancer immunotherapy using checkpoint blockade. Science. 2018;359:1350–5. 10.1126/science.aar4060 PMC7391259 29567705

[2] Gide TN, Pires da Silva I, Quek C, Ferguson PM, Batten M, Shang P, et al. Clinical and molecular heterogeneity in patients with innate resistance to anti-PD-1 +/− anti-CTLA-4 immunotherapy in metastatic melanoma reveals distinct therapeutic targets. Cancers. 2021;13:3186. 10.3390/cancers13133186 PMC8267740 34202352

[3] Havel JJ, Chowell D, Chan TA. The evolving landscape of biomarkers for checkpoint inhibitor immunotherapy. Nature Reviews Cancer. 2019;19:133–50. 10.1038/s41568-019-0116-x PMC6705396 30755690

[4] Cho SF, Anderson KC, Tai YT. Microenvironment is a key determinant of immune checkpoint inhibitor response. Clin Cancer Res. 2022;28:1479–81. 10.1158/1078-0432.CCR-22-0015 35121621

[5] Scheiermann C, Gibbs J, Ince L, Loudon A. Clocking in to immunity. Nat Rev Immunol. 2018;18:423–37. 10.1038/s41577-018-0008-4 29662121

[6] Pick R, Wang C, Zeng Q, Melis Gül Z, Scheiermann C. Circadian rhythms in anticancer immunity: mechanisms and treatment opportunities. Annu Rev Immunol. 2024;42:83–102. 10.1146/annurev-immunol-090122-050842 38941606

[7] Long JE, Drayson MT, Taylor AE, Toellner KM, Lord JM, Phillips AC. Morning vaccination enhances antibody response over afternoon vaccination: A cluster-randomised trial. Vaccine. 2016;34:2679–85. 10.1016/j.vaccine.2016.04.032 PMC4874947 27129425

[8] Phillips AC, Gallagher S, Carroll D, Drayson M. Preliminary evidence that morning vaccination is associated with an enhanced antibody response in men. Psychophysiology. 2008;45:663–6. 10.1111/j.1469-8986.2008.00662.x 18346041

[9] Zhang H, Liu Y, Liu D, Zeng Q, Li L, Zhou Q, et al. Time of day influences immune response to an inactivated vaccine against SARS-CoV-2. Cell Res. 2021;31:1215–7. 10.1038/s41422-021-00541-6 PMC8326654 34341489

[10] Printezi MI, Kilgallen AB, Štibler U, Teske AJ, Cramer MJ, G Sluijter JP, et al. Toxicity and efficacy of chronomodulated chemotherapy: a systematic review [Internet]. Rev Lancet Oncol. 2022;23, Available from: www.thelancet.com/oncology. 10.1016/S1470-2045(21)00639-2 35240088

[11] Shuboni-Mulligan DD, Breton G, Smart DD, Gilbert M, Armstrong TS. Radiation chronotherapy—clinical impact of treatment time-of-day: a systematic review. J Neuro-Oncol. 2019;145:415–27. 10.1007/s11060-019-03332-7 PMC8130840 31729636

[12] Nomura M, Hosokai T, Tamaoki M, Yokoyama A, Matsumoto S, Muto M. Timing of the infusion of nivolumab for patients with recurrent or metastatic squamous cell carcinoma of the esophagus influences its efficacy. Esophagus. 2023;20:722–31. 10.1007/s10388-023-01006-y PMC10123478 37093536

[13] Dizman N, Govindarajan A, Zengin ZB, Meza L, Tripathi N, Sayegh N, et al. Association between time-of-day of immune checkpoint blockade administration and outcomes in metastatic renal cell carcinoma. Clin Genitourin Cancer. 2023;21:530–6. 10.1016/j.clgc.2023.06.004 37495481

[14] Gonçalves L, Gonçalves D, Esteban-Casanelles T, Barroso T, Soares de Pinho I, Lopes-Brás R, et al. Immunotherapy around the clock: impact of infusion timing on stage IV melanoma outcomes. Cells. 2023;12:2068. 10.3390/cells12162068 PMC10453728 37626878

[15] Karaboué A, Collon T, Pavese I, Bodiguel V, Cucherousset J, Zakine E, et al. Time-dependent efficacy of checkpoint inhibitor nivolumab: results from a pilot study in patients with metastatic non-small-cell lung cancer. Cancers. 2022;14:896. 10.3390/cancers14040896 PMC8870559 35205644

[16] Patel JS, Woo Y, Draper A, Jansen CS, Carlisle JW, Innominato PF, et al. Impact of immunotherapy time-of-day infusion on survival and immunologic correlates in patients with metastatic renal cell carcinoma: a multicenter cohort analysis. J Immunother Cancer. 2024;12:e008011. 10.1136/jitc-2023-008011 PMC10966813 38531662

[17] Qian DC, Kleber T, Brammer B, Xu KM, Switchenko JM, Janopaul-Naylor JR, et al. Effect of immunotherapy time-of-day infusion on overall survival among patients with advanced melanoma in the USA (MEMOIR): a propensity score-matched analysis of a single-centre, longitudinal study. Lancet Oncol. 2021;22:1777–86. 10.1016/S1470-2045(21)00546-5 34780711

[18] Rousseau A, Tagliamento M, Auclin E, Aldea M, Frelaut M, Levy A, et al. Clinical outcomes by infusion timing of immune checkpoint inhibitors in patients with advanced non-small cell lung cancer. Eur J Cancer. 2023;182:107–14. 10.1016/j.ejca.2023.01.007 36758475

[19] Yeung C, Kartolo A, Tong J, Hopman W, Baetz T. Association of circadian timing of initial infusions of immune checkpoint inhibitors with survival in advanced melanoma. Immunotherapy. 2023;15:819–26. 10.2217/imt-2022-0139 37191006

[20] Hirata T, Uehara Y, Hakozaki T, Kobayashi T, Terashima Y, Watanabe K, et al. Brief Report: Clinical Outcomes by Infusion Timing of Immune Checkpoint Inhibitors in Patients With Locally Advanced NSCLC. JTO Clin Res Rep. 2024;5:100659. 10.1016/j.jtocrr.2024.100659 PMC11001639 38596201

[21] Huang Z, Karaboué A, Zeng L, Lecoeuvre A, Zhang L, Li XM, et al. Overall survival according to time-of-day of combined immuno-chemotherapy for advanced non-small cell lung cancer: a bicentric bicontinental study. 2025. Available from: www.thelancet.com. 10.1016/j.ebiom.2025.105607 PMC11893323 39983330

[22] Ishizuka Y, Narita Y, Sakakida T, Wakabayashi M, Kodama H, Honda K, et al. Impact of nivolumab monotherapy infusion time-of-day on short- and long-term outcomes in patients with metastatic gastric cancer. Ther Adv Med Oncol. 2025;17:17588359251339921. 10.1177/17588359251339921 PMC12120291 40443962

[23] Zheng Y, Shi F, Sun L, Guo J, Ren T, Ma J. Effect of immune checkpoint inhibitor time-of-day infusion on survival in advanced biliary tract cancer: a propensity score-matched analysis. Front Immunol. 2024;15:1512972. 10.3389/fimmu.2024.1512972 PMC11688298 39744625

[24] Huang Z, Zeng L, Ruan Z, Zeng Q, Yan H, Jiang W, et al. Time-of-day immunochemotherapy in non-small cell lung cancer: a randomized phase 3 trial. Nat Med. 2026. Available from: https://www.nature.com/articles/s41591-025-04181-w. 10.1038/s41591-025-04181-w 41629425

[25] Ascierto PA, Del Vecchio M, Merelli B, Gogas H, Arance AM, Dalle S, et al. Nivolumab for resected stage III or IV melanoma at 9 years. N Engl J Med. 2025. Available from: http://www.nejm.org/doi/10.1056/NEJMoa2504966. 10.1056/NEJMoa2504966 41124198

[26] Patel SP, Kendra K, Grossmann KF, Buchbinder E, Khushalani NI, Eroglu Z, et al. 142P Infusion time of adjuvant PD-1 therapy in high-risk resectable melanoma: Accounting for distance from clinic, scheduling changes over time, and appropriately adjusting *p*-values in SWOG S1404. Immuno-Oncol Technol. 2025;28:101226.

[27] Landré T, Karaboué A, Buchwald ZS, Innominato PF, Qian DC, Assié JB, et al. Effect of immunotherapy-infusion time of day on survival of patients with advanced cancers: a study-level meta-analysis. ESMO Open. 2024;9:102220. 10.1016/j.esmoop.2023.102220 PMC10937202 38232612

[28] Innominato PF, Landré T, Karaboué A, Duchemann B, Buchwald ZS, Zhang YC, et al. Timing of Immunotherapy in Practice: Feasibility and Impact of Morning Checkpoint Inhibitor Administration. JCO Oncol Pract. 2025. Available from: http://ascopubs.org/doi/10.1200/OP-25-00661. 10.1200/OP-25-00661 41397207

[29] Wang C, Zeng Q, Gül ZM, Wang S, Pick R, Cheng P, et al. Circadian tumor infiltration and function of CD8+ T cells dictate immunotherapy efficacy. Cell. 2024;187:2690–2702.e17. 10.1016/j.cell.2024.04.015 38723627

[30] Fortin BM, Pfeiffer SM, Insua-Rodríguez J, Alshetaiwi H, Moshensky A, Song WA, et al. Circadian control of tumor immunosuppression affects efficacy of immune checkpoint blockade. Nat Immunol. 2024;25:1257–69. 10.1038/s41590-024-01859-0 PMC11374317 38806707

[31] Chatterjee M, Turner DC, Felip E, Lena H, Cappuzzo F, Horn L, et al. Systematic evaluation of pembrolizumab dosing in patients with advanced non-small-cell lung cancer. Ann Oncol. 2016;27:1291–8. 10.1093/annonc/mdw174 PMC6276906 27117531

[32] Feng Y, Masson E, Dai D, Parker SM, Berman D, Roy A. Model-based clinical pharmacology profiling of ipilimumab in patients with advanced melanoma. Br J Clin Pharm. 2014;78:106–17. 10.1111/bcp.12323 PMC4168385 24433434

[33] Brahmer JR, Drake CG, Wollner I, Powderly JD, Picus J, Sharfman WH, et al. Phase I study of single-agent anti-programmed death-1 (MDX-1106) in refractory solid tumors: safety, clinical activity, pharmacodynamics, and immunologic correlates. J Clin Oncol. 2010;28:3167–75. 10.1200/JCO.2009.26.7609 PMC4834717 20516446

[34] Karaboué A, Innominato PF, Wreglesworth NI, Duchemann B, Adam R, Lévi FA. Why does circadian timing of administration matter for immune checkpoint inhibitors’ efficacy? Br J Cancer. 2024;131:783–96. 10.1038/s41416-024-02704-9 PMC11369086 38834742

[35] Buchwald Z, Qian DC, Switchenko J, Yushak ML, Lawson DH, Lowe M. The TIME trial: phase II randomized controlled trial of time-of-day-specified immunotherapy for advanced melanoma. J Clin Oncol. 2025;43:TPS9601–TPS9601.

[36] Özdemir BC, Bill R, Okyar A, Scheiermann C, Hayoz S, Olivier T. Chrono-immunotherapy as a low-hanging fruit for cancer treatment? A call for pragmatic randomized clinical trials. J ImmunoTher Cancer. 2025;13:e010644. 10.1136/jitc-2024-010644 PMC11877229 40032603

