# Supplementary material for: Overall survival according to timing of immune checkpoint inhibitors administration in patients with advanced cancer: results from a large single-centre cohort analysis
Source: Br J Cancer. 2026 Jul 18;135(8):1240–6. doi: 10.1038/s41416-026-03494-y (PMC13642331; doi:10.1038/s41416-026-03494-y)
Supplement: Supplementary file 1 [file 41416_2026_3494_MOESM1_ESM.pdf]

Supplementary material

Table of contents

Supplementary figures……………………………………………………….……………………………………….2

Supplementary tables……………………………………………………….………………………….………………….7

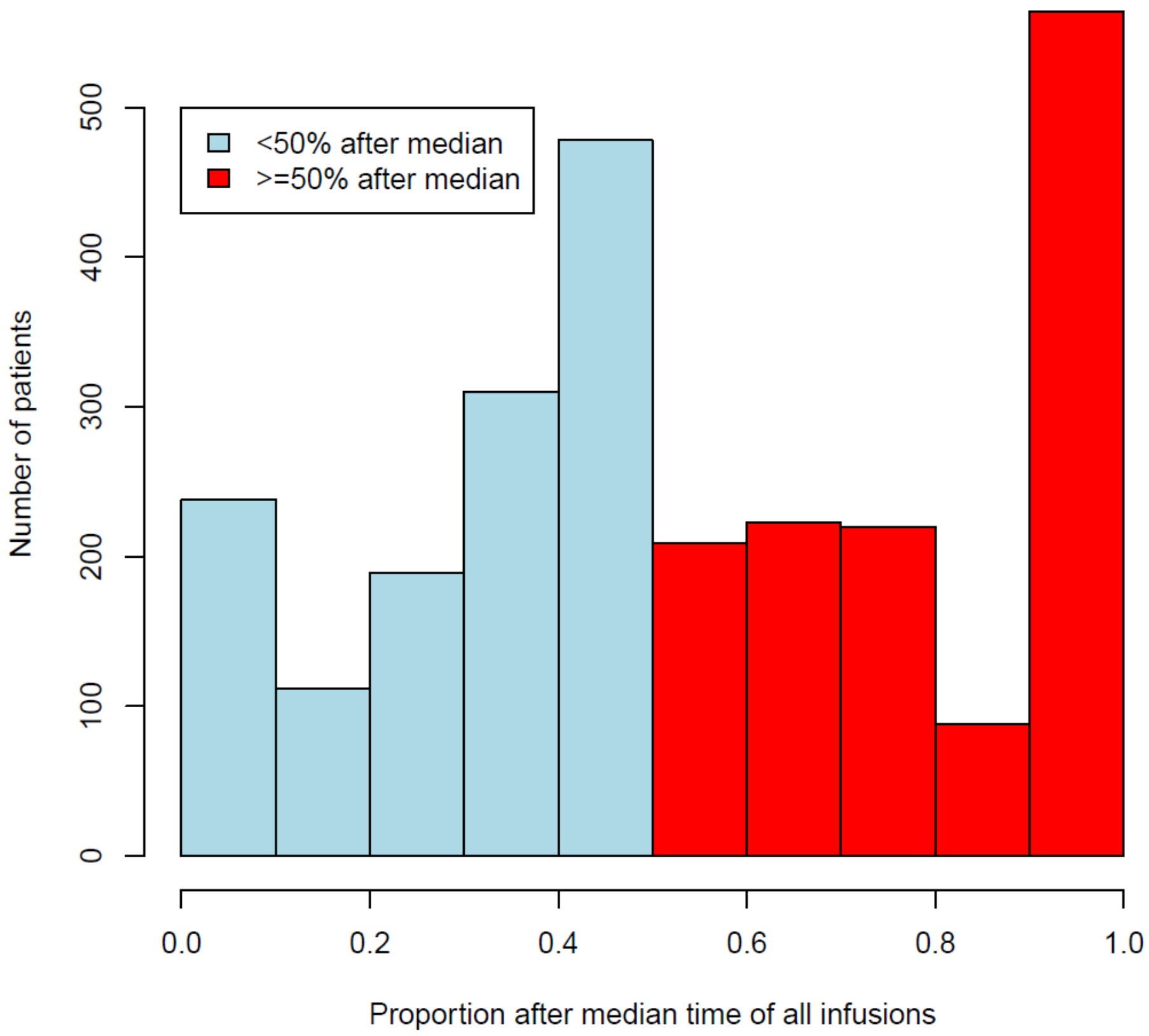

Figure S1: Proportion of treatment administrations after the median time (entire cohort)

(a)

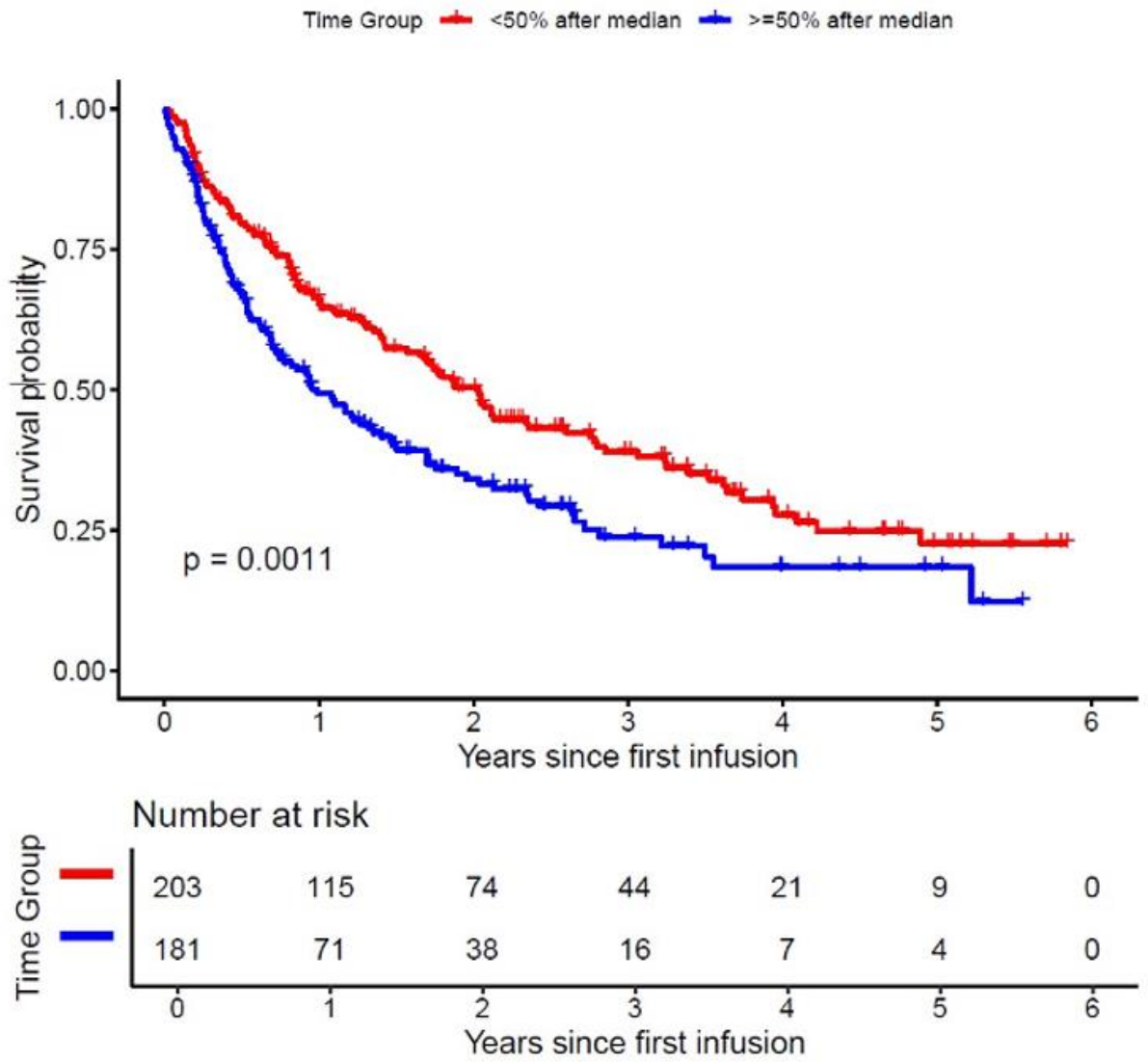

(b)

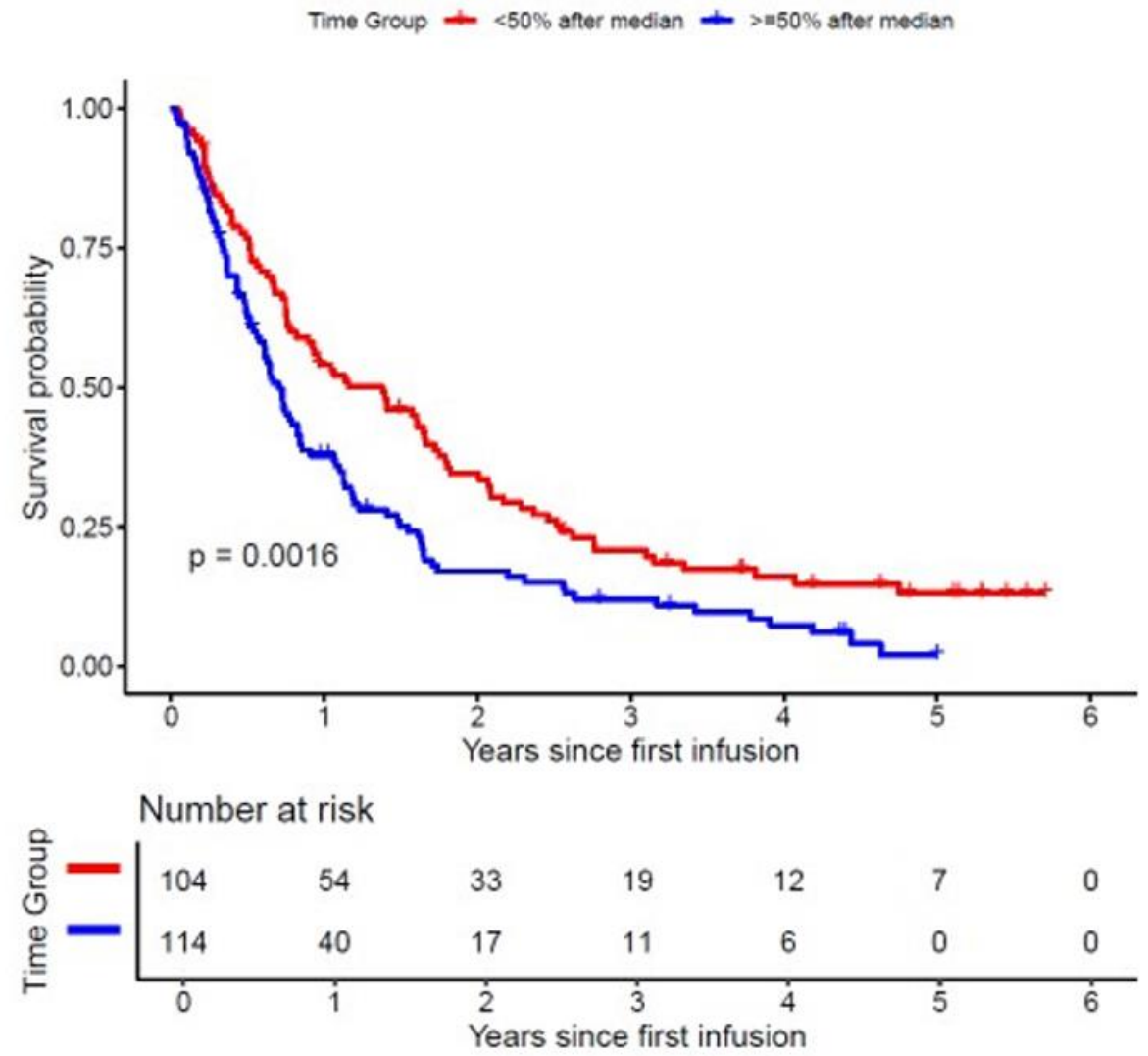

(c)

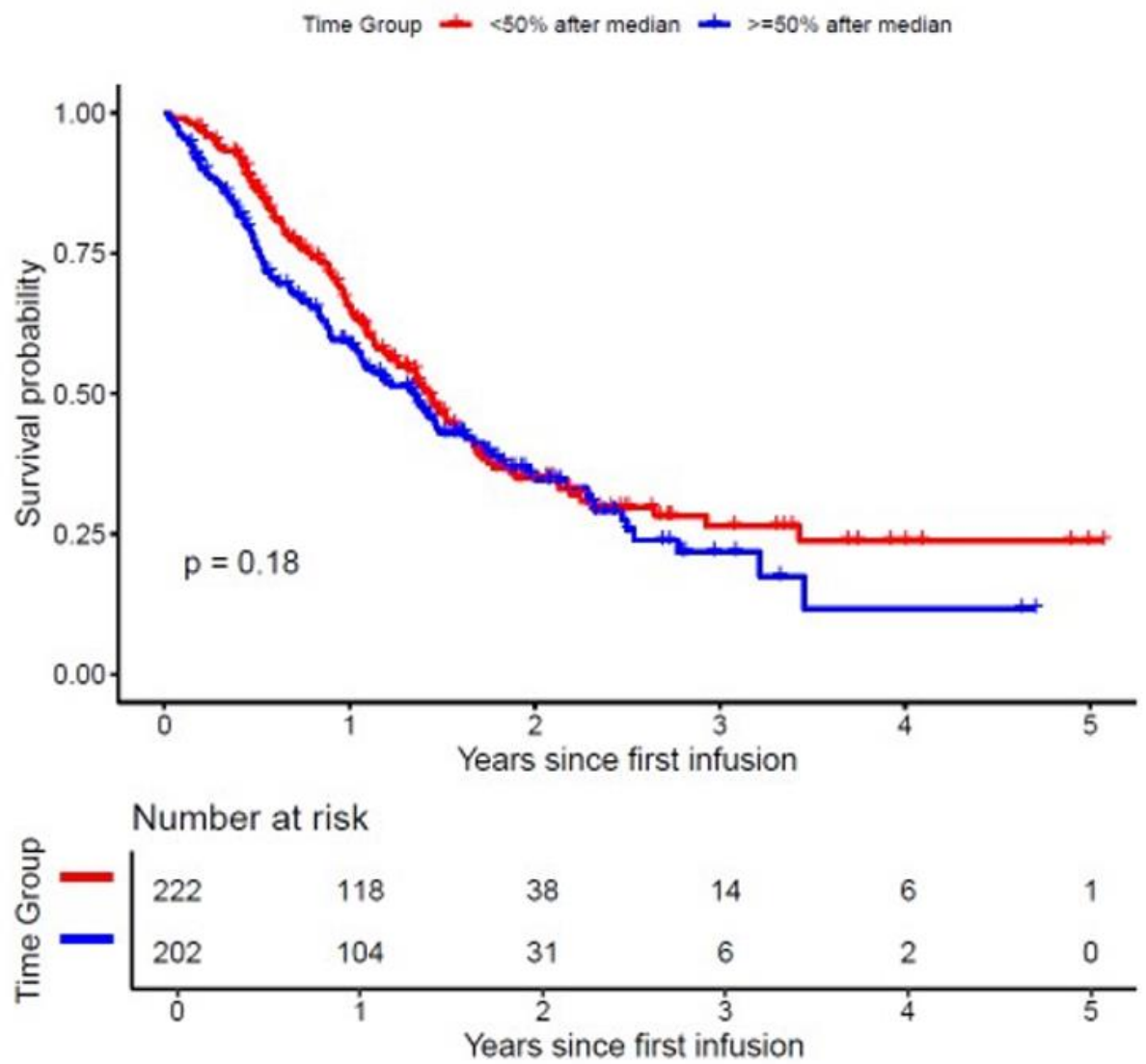

(d)

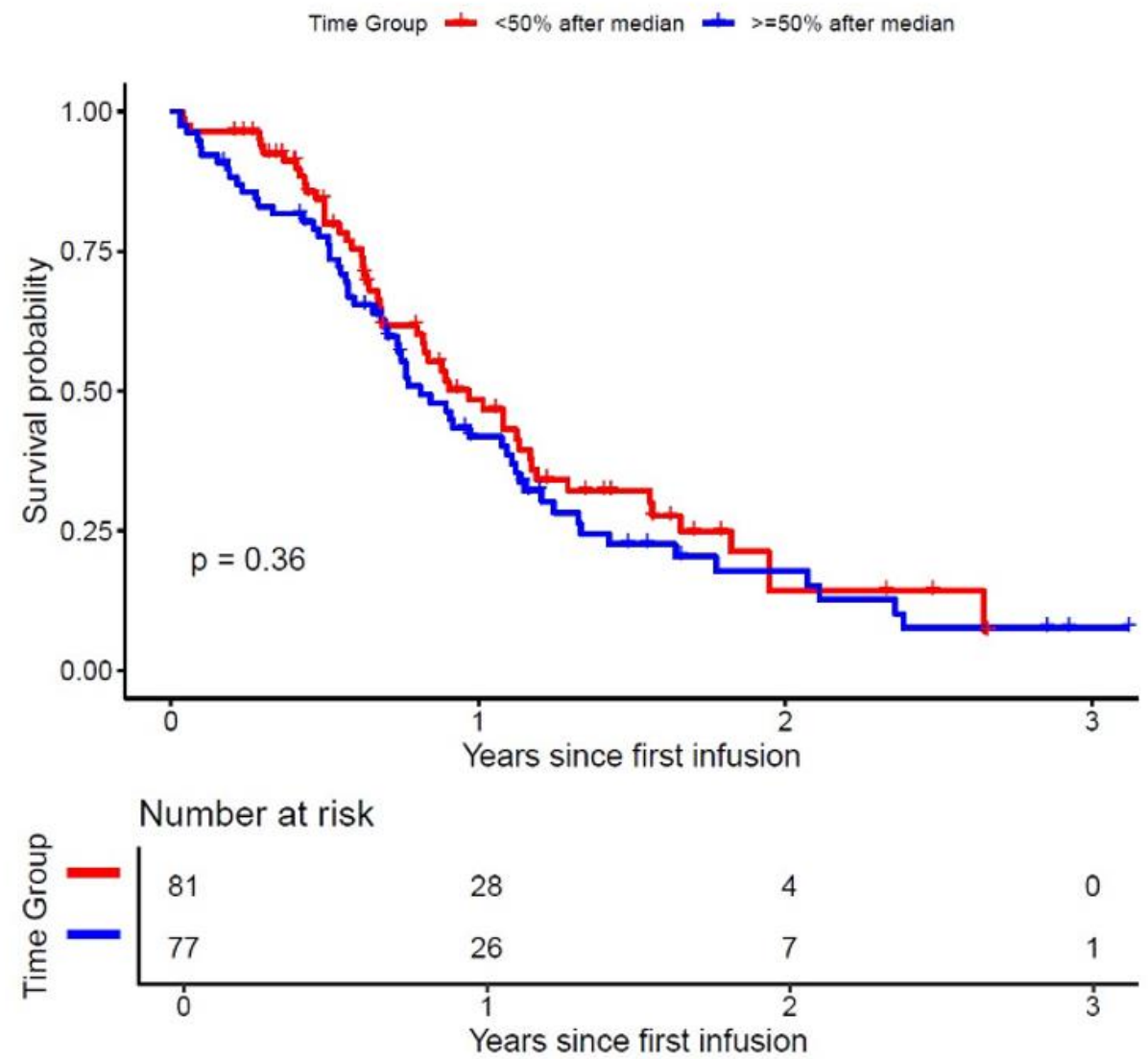

Figure S2: Kaplan-Meier curves for patients who received <50% vs. ≥50% infusions after the median time of infusion (lung cancer). (a) NSCLC – Atezolizumab or pembrolizumab (first line). (b) NSCLC – Atezolizumab or pembrolizumab (second line). (c) NSCLC – Pembrolizumab or atezolizumab with chemotherapy (first line). (d) SCLC – Atezolizumab with chemotherapy. NSCLC=non-small cell lung cancer; SCLC=small cell lung cancer.

(a)

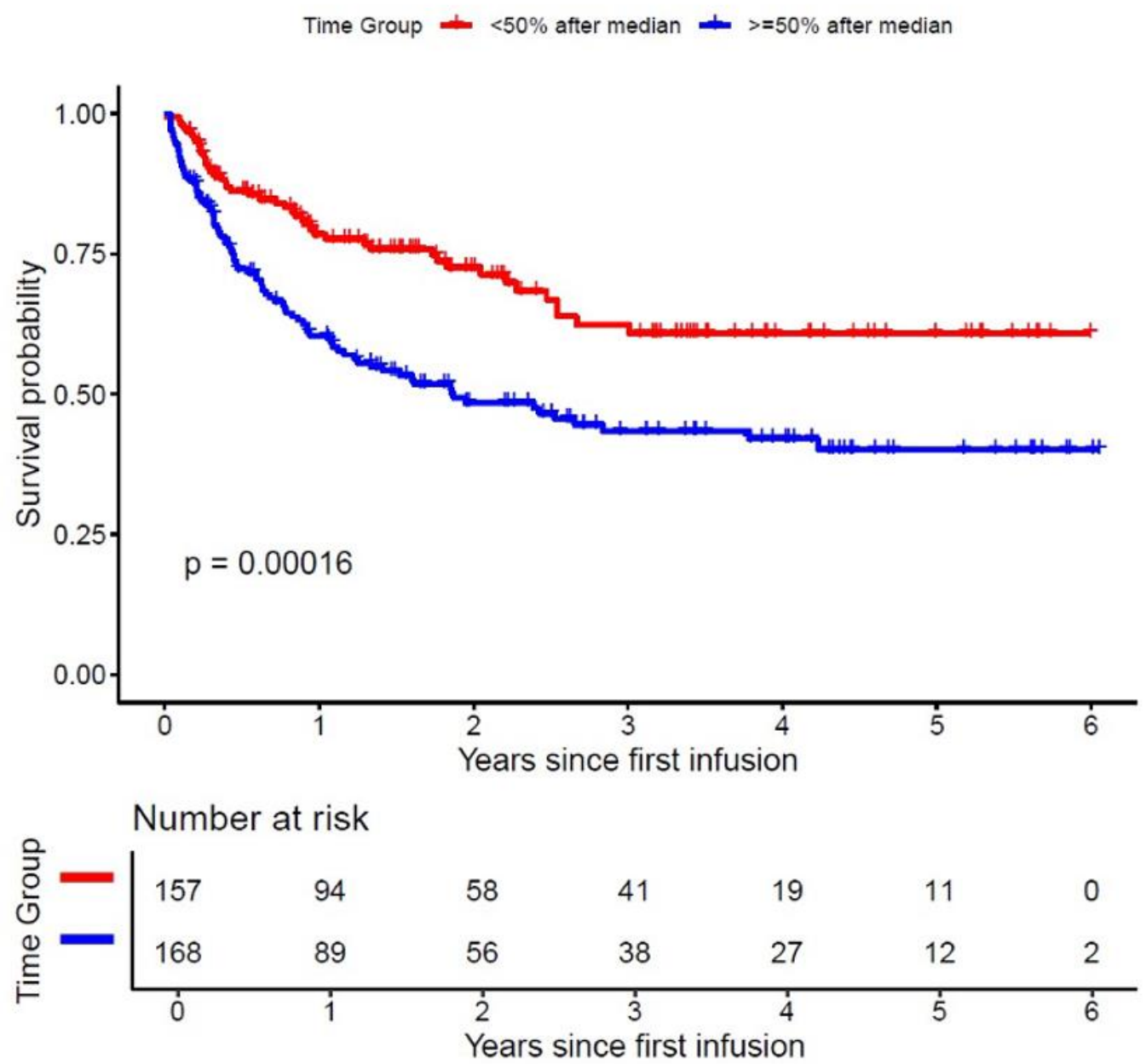

(b)

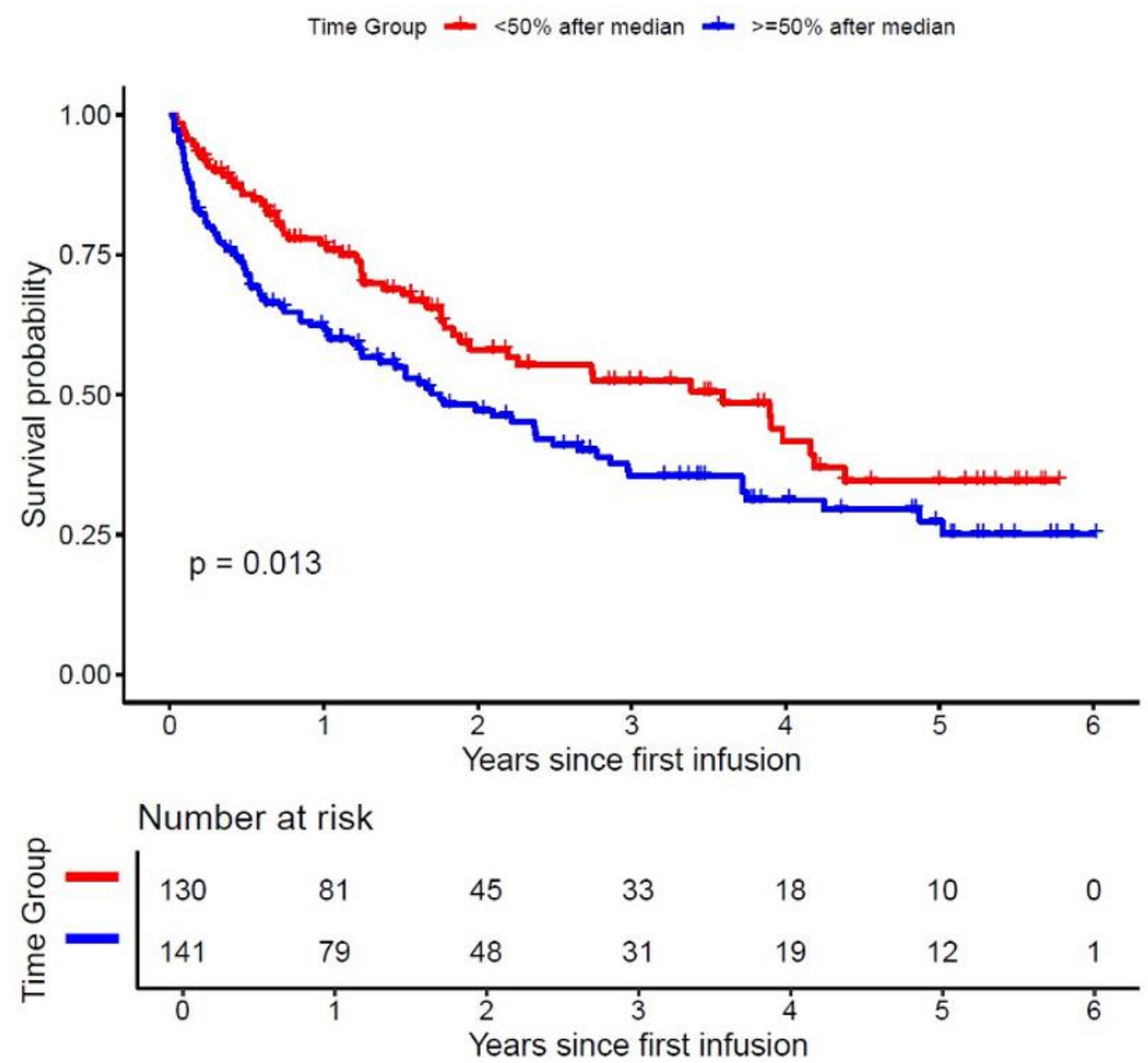

Figure S3: Kaplan-Meier curves for patients who received <50% vs. ≥50% infusions after the median time of infusion (melanoma). (a) Ipilimumab plus nivolumab. (b) Pembrolizumab or nivolumab.

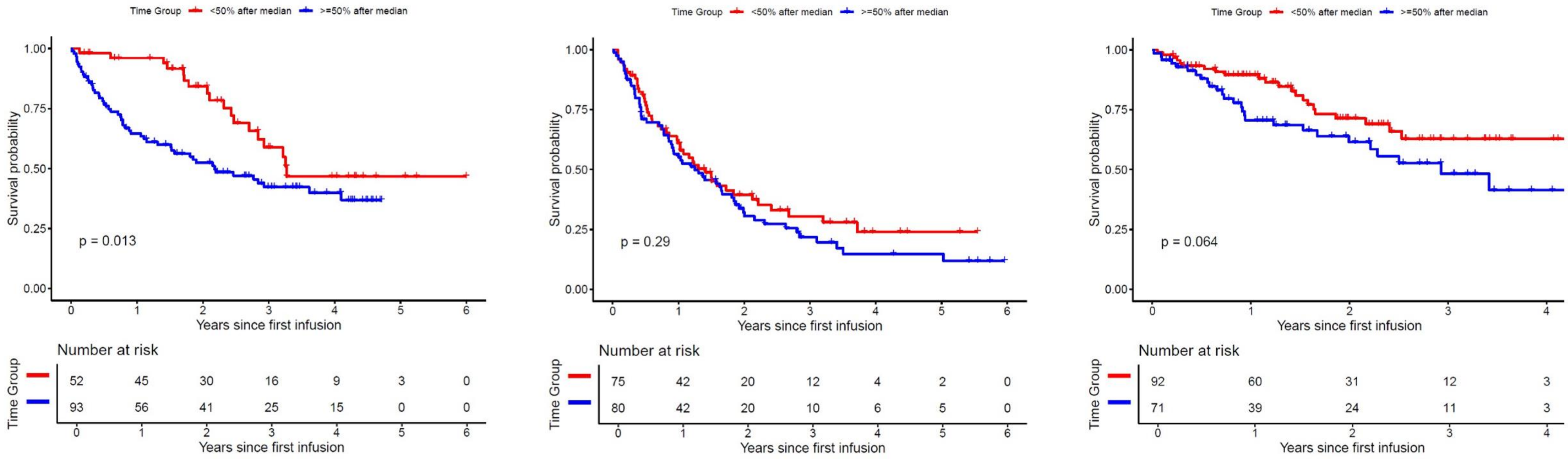

(a) (b) (c)

Figure S4: Kaplan-Meier curves for patients who received <50% vs. ≥50% infusions after the median time of infusion (RCC). (a) Ipilimumab plus nivolumab. (b) Nivolumab. (c) Atezolizumab/pembrolizumab plus TKI. RCC=renal cell carcinoma. TKI=tyrosine kinase inhibitor.

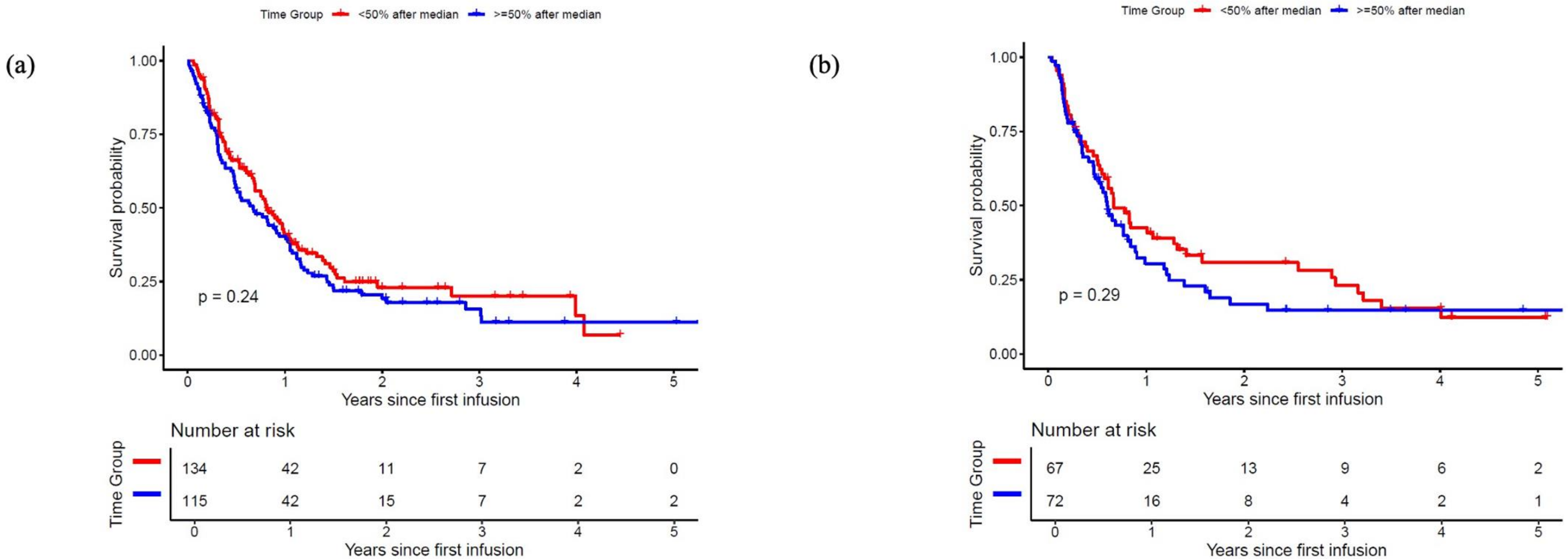

Figure S5: Kaplan-Meier curves for patients who received <50% vs. ≥50% infusions after the median time of infusion (H&N and urothelial cancer). (a) Nivolumab/pembrolizumab for H&N. (b) Atezolizumab/pembrolizumab for urothelial cancer. H&N=head and neck cancer.

Table S1: Overall survival according to timing of administration for the first 3 months of treatment

	Early group		Late group		
	**mOS (95% CI) – months**	**1-year OS (95% CI) – %**	**mOS (95% CI) – months**	**1-year OS (95% CI) – %**	**p value**
All patients	16·9 (15·4-18·4)	60·1 (57·5-62·7)	17·5 (15·0-20·0)	57·7 (54·9-60·8)	0·384
NSCLC					
First line atezolizumab or pembrolizumab	20·2 (14·7-24·8)	59·7 (53·3-66·9)	16·2 (11·3-23·4)	56·2 (48·9-64·7)	0·036
Second line atezolizumab or pembrolizumab	11·6 (9·1-16·9)	49·5 (41·0-59·6)	8·8 (7·3-13·2)	41·4 (32·7-52·2)	0·228
Pembrolizumab or atezolizumab plus chemotherapy	16·3 (13·5-19·1)	64·2 (57·9-71·2)	17·0 (14·1-20·8)	60·1 (53·5-67·7)	0·528
SCLC					
Atezolizumab plus chemotherapy	10·8 (9·6-14·0)	48·4 (38·5-60·9)	9·3 (7·8-13·5)	40·4 (29·5-55·4)	0·301
Melanoma					
Ipilimumab plus nivolumab	NR (27·3-NR)	70·9 (64·5-78·1)	34·0 (23·3-NR)	66·7 (59·0-75·3)	0·34
Nivolumab or pembrolizumab	28·5 (21·1-51·0)	72·1 (64·9-80·0)	26·3 (17·6-35·8)	65·8 (57·8-74·9)	0·202
RCC					
Avelumab or pembrolizumab plus TKI	NR (30·0-NR)	82·6 (74·9-91·1)	41·0 (26·6-NR)	79·0 (69·0-90·4)	0·771
Nivolumab	16·4 (11·8-22·1)	58·7 (49·1-70·3)	15·7 (10·8-23·8)	56·9 (46·0-70·4)	0·819
Ipilimumab plus nivolumab	35·2 (29·5-NR)	79·2 (70·9-88·6)	34·0 (22·2-NR)	70·1 (59·2-83·1)	0·267
H&N					
Nivolumab or pembrolizumab	9·2 (7·7-12·4)	40·9 (33·0-50·7)	9·9 (6·1-12·6)	40·7 (32·3-51·2)	0·809
Urothelial					
Atezolizumab or pembrolizumab	8·0 (6·3-14·8)	40·2 (30·2-53·3)	7·3 (6·2-10·7)	31·7 (21·4-47·0)	0·8

Early group = <50% administrations after the median time. Late group = >/=50% administrations after the median time. OS = overall survival. NSCLC = non-small-cell lung cancer. SCLC = small-cell lung cancer. RCC = renal cell carcinoma. H&N = head and neck cancer. TKI = tyrosine kinase inhibitors. NR = not reached.

Table S2: Multivariable Cox proportional hazard regression of overall survival according to timing of administration for the first three months

	Overall survival HR (95% CI) for late group vs. early group			
	**Standard Cox model**	**p value**	**Time dependent Cox model**	**p value**
All patients	1·09 (0·98-1·20)	0·109	1·14 (1·02-1·26)	0·016
NSCLC				
First line atezolizumab or pembrolizumab	1·24 (0·95-1·60)	0·113	1·17 (0·90-1·53)	0·240
Second line atezolizumab or pembrolizumab	1·15 (0·86-1·54)	0·331	1·21 (0·90-1·61)	0·210
Pembrolizumab or atezolizumab plus chemotherapy	1·07 (0·83-1·38)	0·576	1·02 (0·79-1·32)	0·866
SCLC				
Atezolizumab plus chemotherapy	1·26 (0·86-1·85)	0·228	1·34 (0·90-2·00)	0·147
Melanoma				
Ipilimumab plus nivolumab	1·14 (0·80-1·62)	0·464	1·06 (0·73-1·53)	0·775
Nivolumab or pembrolizumab	1·18 (0·85-1·65)	0·322	1·22 (0·86-1·73)	0·262
RCC				
Avelumab or pembrolizumab plus TKI	1·28 (0·71-2·31)	0·416	0·94 (0·52-1·69)	0·830
Nivolumab	1·03 (0·69-1·54)	0·871	1·24 (0·84-1·83)	0·285
Ipilimumab plus nivolumab	1·36 (0·83-2·23)	0·217	1·47 (0·87-2·49)	0·148
H&N				
Nivolumab or pembrolizumab	0·95 (0·71-1·28)	0·731	0·97 (0·72-1·32)	0·848
Urothelial				
Atezolizumab or pembrolizumab	0·99 (0·67-1·48)	0·970	1·11 (0·74-1·67)	0·599

HR = hazard ratio. Early group = <50% administrations after the median time. Late group = >/=50% administrations after the median time. NSCLC = non-small-cell lung cancer. SCLC = small-cell lung cancer. RCC = renal cell carcinoma. H&N = head and neck cancer. TKI = tyrosine kinase inhibitors.

Table S3: Overall survival according to timing of administration using different cut-offs

Time of day cut-off	Early group		Late group		
	mOS (95% CI) – months	1-year OS (95% CI) – %	mOS (95% CI) – months	1-year OS (95% CI) – %	HR (95% CI) for late vs. early group
10:00h	8·7 (8·0-16·6)	39·3 (27·1-56·9)	17·4 (16·2-18·8)	59·5 (57·5-61·5)	0·70 (0·50-0·98)
11:00h	13·1 (11·1-16·6)	52·7 (46·8-59·5)	17·6 (16·4-19·2)	59·8 (57·7-61·9)	0·91 (0·77-1·07)
12:00h	18·2 (15·7-20·8)	59·9 (56·3-63·7)	16·8 (15·0-18·3)	58·7 (56·5-61·1)	1·12 (1·00-1·25)
13:00h	21·2 (19·8-24·4)	66·1 (63·6-68·7)	12·7 (11·0-14·0)	51·1 (48·2-54·1)	1·51 (1·36-1·67)
14:00h	20·2 (18·8-21·9)	64·7 (62·5-67·0)	9·5 (8·6-10·9)	43·7 (40·1-47·7)	1·73 (1·55-1·93)
15:00h	19·5 (18·0-20·8)	63·5 (61·4-65·6)	5·8 (5·2-7·2)	32·0 (27·4-37·3)	2·22 (1·95-2·53)
16:00h	18·2 (16·9-19·7)	61·2 (59·2-63·2)	4·7 (3·7-6·2)	25·4 (19·2-33·5)	2·50 (2·08-3·01)
17:00h	17·5 (16·5-19·1)	60·1 (58·1-62·1)	2·9 (2·0-4·9)	19·1 (11·4-32·2)	3·19 (2·43-4·19)

OS = overall survival. HR = hazard ratio. Early group = <50% administrations after the selected time of day cut-off. Late group = >/=50% administrations after the selected time of day cut-off.
